# Micronutrients and Anaemia

**DOI:** 10.3329/jhpn.v26i3.1900

**Published:** 2008-09

**Authors:** Kazi M. Jamil, Ahmed Shafiqur Rahman, P.K. Bardhan, Ashraful Islam Khan, Fahima Chowdhury, Shafiqul Alam Sarker, Ali Miraj Khan, Tahmeed Ahmed

**Affiliations:** ICDDR, B, Mohakhali, Dhaka 1212, Bangladesh

**Keywords:** Anaemia, Anaemia, Iron-deficiency, Iodine deficiency, Iron deficiency, Interventions, Micronutrients, Vitamin A deficiency, Bangladesh

## Abstract

Micronutrient deficiencies and anaemia remain as major health concerns for children in Bangladesh. Among the micronutrient interventions, supplementation with vitamin A to children aged less than five years has been the most successful, especially after distribution of vitamin A was combined with National Immunization Days. Although salt sold in Bangladesh is intended to contain iodine, much of the salt does not contain iodine, and iodine deficiency continues to be common. Anaemia similarly is common among all population groups and has shown no sign of improvement even when iron-supplementation programmes have been attempted. It appears that many other causes contribute to anaemia in addition to iron deficiency. Zinc deficiency is a key micronutrient deficiency and is covered in a separate paper because of its importance among new child-health interventions.

## COMBATING VITAMIN A DEFICIENCY IN BANGLADESH

### Introduction

Almost a third of the world's population suffer from micronutrient deficiency, also known as ‘hidden hunger’, which mostly affects those living in developing countries. The public-health importance of vitamin and mineral deficiencies has been underscored through significant investment by national governments and donors in combating micronutrient deficiency to achieve “Millennium Development Goals” relating to mother and child health. In the following section, the deficiency of vitamin A has been reviewed in the context of Bangladesh. Beginning from historical perspectives of the control programmes, the most recent and ongoing activities of the Government have been highlighted with emphasis on successful interventions that need to be continued for sustained outcome. Attempts have been made to identify weaknesses of the programmes with recommendations on how to overcome them with reference to other successful programmes in similar settings.

- The vitamin A programmes of the past have been extremely effective and need to be sustained.
- Despite the legal requirement of iodized salt, a large proportion of the salt does not contain sufficient iodine, and a large proportion of the population is iodine-deficient.
- Anaemia rates remain high. Iron supplements and/or fortificants may help, but much of the anaemia is not due to iron deficiency.
- Zinc is a life-saver, and programmes for its use are critically needed.

### Vitamin A deficiency situation in Bangladesh

Vitamin A deficiency, the leading cause of preventable blindness in children, increases the risk of disease and death from severe infections. Vitamin A deficiency has long been identified as a serious public-health problem in Bangladesh, especially since the first national nutrition survey conducted in the then East Pakistan in 1962–1964 (1). In this survey, 0.2% of the total population was found to have Bitot's spots, and 4.2% had conjunctival xerosis. A historical perspective of vitamin A deficiency assessed by the nationwide nutrition surveys are summarized in Table 1.

**Table 1:** Prevalence of vitamin A deficiency among the general population during 1962–1982 (based on clinical signs)

Study period	Nightblindness (%)	Bitot's spots (%)	Conjunctival xerosis (%)	Survey conducted by	Reference no.
1962–1964	NA	0.2	4.2	Government of East Pakistan	1
1975–1976	1	0.6	11.7	Government of Bangladesh	2
1981–1982	0.2	0.3	15.3	Government of Bangladesh	3

NA=Not available

With the decline in the prevalence of severe clinical manifestations of vitamin A deficiency, nightblindness was found to be the most useful indicator for the assessment of vitamin A deficiency. In addition, the subsequent surveys focused on more vulnerable groups, especially preschool children and mothers, rather than the whole population. Data on nightblindness in preschool children and mothers since 1982 are summarized in Table 2.

**Table 2:** Prevalence of nightblindness among preschool children of Bangladesh

Study period	Children aged <6 years (%)	Mothers (%)	Survey conducted by	Reference no.
1982–1983	3.6 (R) 2.8 (U)	-	HKI/IPHN	4
1989	1.78 (R)	-	IPHN/UNICEF	5
1995	1.1	-	HKI	6
1997	0.62 (R)	2.2 (R)	HKI	7
2003	0.19 (R) 0.06 (U)	0.65 (R) 0.47 (U)	IPHN/HKI	8
2004	0.07 (R)	0.39 (R)	IPHN/HKI	9

HKI=Helen Keller International; IPHN=Institute of Public Health Nutrition; R=Rural; U=Urban; UNICEF=United Nations Children's Fund

Vitamin A status has been assessed by clinical examination and identification of signs of deficiency in the national nutrition surveys. However, studies conducted by other investigators have used biochemical indicators of vitamin A deficiency among various population groups. Table 3 provides data on the prevalence of vitamin A deficiency among preschool children based on clinical signs and symptoms. Table 4 provides data on retinol levels in a similar age-group. A summary of data on serum retinol levels in populations of different demographic groups in Bangladesh is given in Table 5.

**Table 3:** Prevalence of vitamin A deficiency among preschool children of Bangladesh (based on clinical signs and symptoms)

Study period	Setting	Age-group	Sample size	Nightblindness (%)	Bitot's spot (%)	Corneal xerosis (%)	Reference no.
1975–1976	Community (rural)	0-4 year(s)	469	1.3	0.0	9.8	2
1981–1982	Community (rural)	0-4 year(s)	514	0.6	0.3	17.6	3
1982–1983	Community (rural and urban slums)	3-71 months	18,660 (R) 3,675 (U)	3.8 (R) 2.8 (U)	0.9 (R) 1.6 (U)	2.0 (R) 2.5 (U)	4
1981–1985	Hospital (periurban)	<5 years	22,407	1.0	2.0	0.2	11
1983–1985	Hospital (urban)	6-36 months	2,687	1.71	0.56	1.15	12
1989	Community (rural)	6-71 months	4,611	1.78	NA	NA	5
1996	Community (rural)	6-59 months	16,140	0.60	NA	NA	13

NA=Not available; R=Rural; U=Urban

**Table 4:** Serum retinol levels in preschool children of Bangladesh

Study period	Setting	Age-group	Sample Size	Mean serum retinol (μmol/L)	Serum retinol level (μmol/L)			Reference no.
					<0.35 (%)	<0.70 (%)	<1.05 (%)	
NA	Hospital and community	1-6 year(s)	95	0.49	20	NA	NA	17
1980–1981*	Hospital (urban)	3-9 years	13	0.8	NA	NA	NA	18
NA	Hospital (urban)	1-10 year(s)	30	NA	66	100	NA	19
1987	Hospital (rural and urban)	1-5 year(s)	36	0.76† 0.88‡	NA	NA	NA	20
NA	Hospital (urban)	6-17 weeks	183	0.43† 0.47‡	35	87	100	21
1994	Hospital (urban)	2-11 months	85	0.66	18	56	NA	22

*Information about the study period in some cases has been obtained from the authors through personal communication;

†Values at baseline: Treatment group;

‡Control group

NA=Not available

**Table 5:** Serum retinol levels in population of different demographic groups in Bangladesh

Study period	Study population	Setting	Age-group (years)	Sample size	Mean serum retinol (μmol/L)	Serum retinol level (μmol/L)			Reference no.
						<0.35 (%)	<0.70 (%)	<1.05 (%)	
1992	Adolescent girls	School (urban)	12-15	225	>1.05	NA	NA	11	23
1996	Adolescent female workers	Factory (urban)	12-19	388	1.04	0.5	14	56	24
NA	Female workers	Factory (urban)	20-35	63	1.19	9	56	NA	25
NA	Women at delivery	Hospital (urban)	15-40	85	NA	NA	5	30	26
1994	Women at delivery	Hospital (urban)	20-30	151	1.33	NA	NA	30	27
NA	Women at delivery	Hospital (urban)	16-35	50	>1.05	NA	NA	NA	28

NA=Not available

### Vitamin A capsule programmes

The Government of Bangladesh started the supplementation of vitamin A capsules in 1973 under the National Blindness Prevention Programme, and it remains one of the most successful programmes (10). A detailed account of the coverage of the programme is enumerated in Table 6 and 7.

**Table 6:** Supplementation of vitamin A capsule to preschool children in Bangladesh during 1973–1995

Year	Dosage of vitamin A given to different age-groups	Coverage (%)	Reference no.
1973	Children aged 12-71 months: 200,000 IU Children aged <12 months: 100,000 IU	NA	10
1982–1983	Children aged <12 months: 100,000 IU	45 (rural)	29
1987–1988	Children aged <12 months: 100,000 IU	37 (rural)	5
1989	Children aged <12 months: 100,000 IU	35 (rural)	5
1994	Children aged 6 months: 50,000 IU 2nd dose at 10-14 weeks: 50,000 IU 3rd dose at 36 weeks: 50,000 IU Children aged 12-71 months: 2,00,000 IU every six months	45	29
June 1995	Children aged 12-71 months: 2,00,000 IU every six months	87 (rural)	13
December 1995	Children aged 12-71 months: 2,00,000 IU every six months	83.6 (rural) 73.7 (urban)	13

NA=Not available

**Table 7:** Coverage of vitamin A capsule supplementation from BINP data, 1995–2003

Target groups who received vitamin A capsules	Baseline (1995)	Mid-term (1998)	Endline (2003)
Vitamin A capsule given to children aged >11 months	Project (n=124): 39.0% Control (n=36): 41.7%	Project (n=2,121): 84.1% Control (n=675): 72.3%	Project (n=1,732): 72.3% Control (n=590): 61.0%
Postpartum vitamin A capsule given to mother after the last pregnancy	NA	Project (n=4,465): 56.3% Control (n=1,502): 10.0%	Project (n=3,729): 65.0% Control (n=1,238): 13.0%

BINP=Bangladesh Integrated Nutrition Project; NA=Not available

In the mid-1990s, supplementation of vitamin A to children aged less than 12 months was integrated into the Expanded Programme on Immunization (EPI), resulting in an increased coverage for this group. In 1995, vitamin A administration among 1-6 year(s) old children was integrated with the National Immunization Day (NID) which also succeeded in increasing the coverage for preschool children. Subsequently, the Government initiated the week-long nationwide mobilization campaign—‘National Vitamin A Week’—for distribution of vitamin A to children aged 12-71 months. To include mothers, postpartum vitamin A supplementation was then started by the Government with the support of United Nations Children's Fund. Later, the Bangladesh Integrated Nutrition Project (BINP) started distribution of vitamin A capsules among mothers within 14 days of delivery and reported achievement of 65% coverage in 2003 (14). However, the Baseline Survey of the National Nutrition Programme, conducted by ICDDR, B and National Institute of Population Research and Training reported postpartum vitamin A capsule-distribution coverage of only 8% (15).

### Dietary intake of vitamin A

All the three successive national nutrition surveys (1962–1964, 1975–1976, and 1981–1982) in Bangladesh reported an insufficient intake of vitamin A. In the 1962–1964 survey, the average intake was 93% of the recommended intake which dropped to 36% in 1975–1976. In the 1981–1982 survey, the situation changed very little with an intake of about 39% of recommended dietary allowance. In rural Bangladesh, about 90% of dietary vitamin A came from fruits and vegetables (3).

**Increasing vitamin A in diet**

- Improve food diversification, including greens and fruits.
- Introduce new foods, such as orange sweet potatoes.
- Introduce fortified foods where possible, e.g. cooking oil or *atta.*

**Recommendations for vitamin A programmes in Bangladesh**

- Continue distribution of vitamin A capsules to preschool children.
- Although dependent on external donor support, the vitamin A capsule programme is highly effective and needs continuation by the Government of Bangladesh.
- Improve coverage of postpartum vitamin A capsule distribution—it is lagging.
- Include supplementation of vitamin A capsules to pregnant women in the National Nutrition Programme. A weekly supplement of up to 25,000 IU (8,500 μg) is an alternative to daily supplementation.

In 1988, Helen Keller International (HKI) initiated home-gardening in North Bengal in an attempt to improve the intake of vitamin A by the population. Home-gardening activities rapidly expanded to cover 60-70% of upazilas by 1998. Bloem *et al.* claimed that production of pro-vitamin A-rich fruits and vegetables in the homestead might provide a valuable contribution to vitamin A intake in the communities where alternative sources of vitamin A are scarce (16). More recent programmes by HKI included animal food production, e.g. poultry, in addition to home-gardening. A programme evaluation using direct indicators, such as measurement of serum retinol or assessment of vitamin A pool size when possible, will be needed to determine the impact of this approach in reducing vitamin A deficiency in the population (30).

### Recommendations for improving vitamin A status

Nutrition education should be provided through government outreach centres to increase the intake of vitamin A-rich foods by increasing those from animal origin together with adequate intakes of fruits and vegetables. Cooking techniques need to preserve the bioavailability of vitamin A in foods (30). Studies conducted in Africa have shown that consumption of orange-fleshed sweet potato (OFSP), which is rich in beta-carotene, can effectively reduce vitamin A deficiency in children (31). The same variety of OFSP is being grown in Bangladesh at research centres of Bangladesh Agricultural Research Institute. The low cost of production and high growth potential of OFSP make it an ideal food, meeting the requirements of vitamin A and total energy. Continued research is needed to determine if this can be successfully introduced into diets in Bangladesh and to determine the extent to which requirements of vitamin A can be met with a staple food, like this one which is rich in vitamin A.

Fortification of food is yet another effective means to increase the intake of vitamin A by the entire population. At present, only one private company in Bangladesh produces edible oil fortified with vitamin A. Government efforts must be made to promote food fortification to combat vitamin A deficiency in Bangladesh. Alternatively, micronutrient ‘sprinkles’ can be added to foods in the home, but additional operations research is needed to determine if this newer technology will be acceptable in Bangladesh (32).

## IODINE DEFICIENCY DISORDERS IN BANGLADESH

### Introduction

Iodine deficiency is considered to be the most common preventable cause of mental disorders in the world today, having manifestations at different stages of human life. A large proportion of people with severe iodine deficiency are women of reproductive age, who are at a higher risk of pregnancy-related problems, including abortion, stillbirth, low-birthweight infants, brain damage or cretinism in infants even before birth, and lower chance of survival. Iodine deficiency can cause goitre and brain damage in neonates whereas manifestations in children include goitre, loss of energy, impaired school performance, and retarded physical development. In adults, iodine deficiency can lead to goitre and related complications, loss of energy, and impaired mental function. According to a 1990 WHO report, some 26 million people suffer from brain damage associated with iodine deficiency disorder, which includes six million cretins (33). All these have resulted in a growing awareness of the problem all over the world.

### Iodine deficiency and goitre in Bangladesh

Results of surveys conducted since the 1960s have shown that high levels of iodine deficiency are prevalent in Bangladesh. The Nutrition Survey of East Pakistan 1962–1964 reported a goitre rate of 28.9% in former East Pakistan, now Bangladesh (1). The 1981–1982 National Goitre Prevalence Survey reported levels of iodine deficiency disorder nationwide, with the goitre rate at 10.5% (34). This result was, however, criticized because the health officers and workers who were assigned to identify goitre cases were not adequately trained. The National Iodine Deficiency Disorder Survey 1993 revealed a goitre rate of 47.1% (35). Another survey, using the ‘EPI-30 cluster’-sampling methodology, found a prevalence of cretinism of 0.5%; 69% of subjects had low urinary concentrations of iodine (urinary iodine excretion [UIE] <10 mg/dL). Women and children were more affected than men, in terms of prevalence of both goitre and UIE. The presence of widespread severe iodine deficiency in all ecological zones indicates that the country as a whole is an iodine-deficient region (36).

### Salt iodization in Bangladesh

To combat iodine deficiency disorder, the Government of Bangladesh, in 1989, passed the Iodine Deficiency Disease Prevention Act. The Act proclaimed universal iodization of edible salt for human and animal consumption and included prevention, enforcement, and education efforts. Under this act, the Bangladesh Council of Scientific and Industrial Research (BCSIR) and other institutions would be responsible for monitoring the quality of iodized salt manufactured and sold from that time onwards. Most salt-crushing units (which produce pure white sea salt from impure coloured products of salt croppers) have been provided with iodization equipment, and UNICEF supplies the iodizing agent—potassium iodate—free of charge. Despite this law and this assistance, much of the salt used by the people is not iodized.

A survey in 1995 showed that only 30% of iodized salt manufactured in Bangladesh contained an acceptable level of iodine (37). Surprisingly, 10% of commercial brands contained no iodine at all. Only 30% of producers were using the recommended level of iodine, 10% were using mixtures of fortifying agents, and 10% were not using any iodine at all. Another survey conducted with UNICEF support in 1997 showed that the situation had not improved (36). This survey found that only 57% of salt factories with iodization facilities were in regular production, 7% produced iodized salts only irregularly, and 36% were closed. Of 379 samples collected from 138 factories, only 5% contained adequate amounts of iodine, 46% contained too little, 1% contained no iodine at all, and 49% contained too much; some contained significantly more than it should, i.e. up to 20 times the recommended amount of iodine. Of 1,104 samples collected from retail outlets, 7% contained no iodine, and only one contained the recommended amount whereas 44% contained too little, and 56% contained a very large excess. Another cross-sectional study conducted in a coastal area in southern Bangladesh, during 1997–1998, comprising 21,190 households revealed that only 1.9% of the households used iodized salt in daily cooking (38). In the Baseline Survey of the National Nutrition Programme in 2004, 39.5% of households were consuming table salt containing an inadequate concentration of iodine (<15 ppm) (15).

The barriers limiting the use of iodized salt include the wide availability of coarse salt, lack of knowledge about the link between iodized salt and iodine deficiency disorders, and the high cost of iodized salt. These data show that the salt-iodization programme in Bangladesh is not making headway. The reasons may include: lack of quality-control measures in production units, lack of skills among production personnel, and failure on the part of the government regulatory agencies.

## ANAEMIA: A PUBLIC-HEALTH PROBLEM IN BANGLADESH

### Introduction

Anaemia, a major public-health problem, was identified about four decades ago in Bangladesh. In the following section, the trend in the prevalence of anaemia among different age- and sex-groups is presented. Information was collected from national nutrition surveys and from intervention and observational studies. Besides, the prevalence of iron deficiency was also documented from available sources. This section reviews the consequences of anaemia on health outcomes in children and women. The section also highlights on the aetiological factors causing anaemia and reviews the strategies that can be adopted to prevent the magnitude and extent of the condition.

### Prevalence of anaemia in Bangladesh

Anaemia is defined as a reduction in the oxygen-carrying capacity of blood. It is observed by reduced levels of haemoglobin concentration and red cell mass (haematocrit). At an individual level, however, anaemia is said to exist when haemoglobin concentration falls below a threshold: standard deviation of ±2 below the median for a healthy population of the same age, sex, and stage of pregnancy (39). The criteria for assessing the magnitude of the anaemia problem in relation to public-health significance (40) and the recommended cut-off points of haemoglobin levels for defining the presence of nutritional anaemia (41) are shown in Table 8.

**Table 8:** Recommended cut-off points of haemoglobin levels to define anaemia in population groups (41) and criteria for assessing the magnitude of the anaemia problem in relation to public-health significance (40)

Group	Cut-off points of haemoglobin level (g/L)	Public-health significance		
		Category	Mild-moderate anaemia (Hb 70-109 g/L) (%)	Severe anaemia (Hb <70 g/L) (%)
Children		Severe	>40.0	>10.0
6 months-5 years	<110.0			
5-11 years	<115.0			
12-13 years	<120.0			
Women		Moderate	20.0-39.9	5.0-19.9
Non-pregnant	<120.0			
Pregnant	<110.0			
Men	<120.0	Mild	1.0-9.9	0.1-0.9

Although anaemia has been recognized as a public-health problem for many years, there has been little progress towards improvement and the global prevalence of anaemia remains unacceptably high. It has been estimated that around two billion people in the world are anaemic, mostly in the lower-income countries of Africa and Asia. In Bangladesh, anaemia is common among all age-groups, and both sexes are affected, especially children and women—both pregnant and non-pregnant.

Although there are many causes of anaemia, three main aetiologic categories are of concern in developing countries. These are: (a) nutritional deficiencies, (b) chronic infection, and (c) haemoglobinopathies.

Over the last four decades, data on the prevalence of anaemia have been gathered from national nutrition surveys conducted during this period and from a number of studies, which have been carried out to investigate the prevalence of anaemia, or from baseline information of a number of intervention or observational studies. Many of these studies have been done with small numbers and/or samples that were not representative of the populations of the country. However, all information is important in indicating the magnitude and trend of this public-health problem in Bangladesh.

The anaemia-prevalence data in preschool children are summarized in Table 9. All the surveys indicated a high prevalence of anaemia, without any trend for improvement. Table 10 shows similar information for older children. Overall, it would seem that rates of anaemia in this age-group may be decreasing, although the rates are still very high at 30-40% for school-age children. The rates for girls are slightly higher than for boys. For pregnant women, the available prevalence data are summarized in Table 11. The general findings of these surveys suggest that nearly half of the pregnant women have anaemia. Somewhat surprisingly, anaemia is also highly prevalent in non-pregnant women and in adult males in Bangladesh. The prevalence data on anaemia for adult males and non-pregnant women are summarized in Table 12. Most of these surveys showed rates exceeding 50% even in men.

**Table 9:** Summary of prevalence data on anaemia among preschool children in Bangladesh

Study period	Area	Setting	Age-group	Sample size	Prevalence of anaemia (%) *	Reference no.
1975–1976	R	C	0-4 year(s)	163	82.0	2
1981–1982	R	C	0-4 year(s)	421	73.0	3
1995–1996	R U	C	0-4 year(s)	616 169	69.5 38.5	42
1997–1998	R	C	6-59 months	1,199	52.7	43
2001	R	C	6-59 months	1,148	48.3	44
2003	U CHT	Slum/non-slum C	6-59 months	861 462	56 62	45
2004	R	C	6-59 months	1,227	67.9	46,47

* Defined by a haemoglobin level of <110 g/L; C=Community; CHT=Chittagong Hill Tracts; R=Rural; U=Urban

**Table 10:** Summary of prevalence data on anaemia among school-age and adolescent children in Bangladesh

Study period	Area	Setting	Age-group (years)	Sex	Sample size	Prevalence of anaemia (%) *	Reference no.
1962–1964	R	C	5-14	M F	88 48	46.0 50.0	1
1975–1976	R	C	5-14	M F	463 384	74.0 75.0	2
1981–1982	R	C	5-14	M F	435 383	74.0 73.0	3
1992	U	S	12-15	M and F	225	22.0	23
1995–1996	R U	C	5-14	M and F	1,346 407	80.4 70.5	42
1996	U	Factory	11-19	M and F	388	44.0	24
1996	R	S	7-10	M and F	400	51.5†	48
1996	Periurban	S	11-16	M and F	548	27.0	49
1997–1998	R	C	6-11 11-16	M and F	328 196	38.4† 43.0	43
2001	R	C	5-11 12-14 15-19	M and F F	1,734 412 189	33.5† 35 30	44
2002	R	C	6-15	M and F	334	41.0	50
2003	U CHT	Slum/ non-slum C	13-19	M and F	1,341 631	23.0 43.0	45
2004	R	C	13-19	M F	648 661	30.9 39.7	46,47

*Defined by a haemoglobin level of <120 g/L;

†Defined by a haemoglobin level of <115 g/L; Hb <130 g/L males; C=Community;

CHT=Chittagong Hill Tracts; F=Female; M=Male; R=Rural; S=School; U=Urban

**Table 11:** Summary of prevalence data on anaemia among pregnant women in Bangladesh

Study period	Area	Setting	Gestation period	Sample size	Prevalence of anaemia (%)*	Reference no.
1962–1964	R	C	NA	135	59.5†	1
1975–1976	R	C	NA	174	50.0	2
1981–1982	R	C	NA	279	47.0	3
1990	U	MC	12-16 weeks 24-28 weeks At delivery	209 89 28	21.0 32.6 43.0	51
1993	U	MC	At delivery	151	20.0	27
1995–1996	R and U	C	NA	70 (R) 15 (U)	60.0 (R) 53.0 (U)	42
1997	R	ANCC	2^nd^ trimester	214	50.0	52
1997–1998	R	C	NA	120	49.2	43
1998	U	MC	20-32 weeks	389	39.0	53
2001–2002	R	C	1^st^ trimester	350	47.7¶	54
2003	U CHT	Slum/nonslum C	NA	500 368	41 49	45
2004	R	C	NA	102	38.8	46,47

*Defined by a haemoglobin level of <110 g/L;

†Haemoglobin level <120 g/L;

¶Haemoglobin level <80 g/L; ANCC=Antenatal care centre; C=Community; CHT=Chittagong Hill Tracts; MC=Maternity clinic; NA=Not available;

R=Rural; U=Urban

**Table 12:** Summary of prevalence data on anaemia among adult males and non-pregnant females in Bangladesh

Study period	Area	Setting	Sex	Sample size	Sampling design	Haemoglobin method	Prevalence of anemia (%)*	Reference no.
1962–1964	R	C	M F	630 177	Multi-stage random	Cyanomethaemoglobin	69.0† 55.0	1
1975–1976	R	C	M F	590 437	Two-stage Systematic Random	Cyanomethaemoglobin	62.0 70.0	2
1981–1982	R	C	M F	628 442	Two-stage Systematic Random	Cyanomethaemoglobin	60.0 74.0	3
1995–1996	R and U	C	M F	1,601 (R) 1,322 (R)	Systematic Random	Cyanomethaemoglobin	68.0 81.0	42
1996	R	C	F	179	Selected	Cyanomethaemoglobin	73.0	58
1997–1998	R	C	F	1,082	Random	HaemoCue	45.0	43
2003	U CHT	Slum/non-slum	F	884 419	Random selection from clusters	HaemoCue	34.0 39.0	45
2004	R	C	F	1,388	Muti-stage cluster	HaemoCue	46.0	46,47

Defined by a haemoglobin level of <130 g/L for males and <120 g/L for females;

Haemoglobin level <139 g/L; C=Community; CHT=Chittagong Hill Tracts; F=Female; M=Male; R=Rural; U=Urban

### Prevalence of iron deficiency in Bangladesh

Many equate anaemia with iron deficiency. While iron is certainly crucial for normal levels of haemoglobin, iron deficiency is not the only cause of anaemia. Several studies have been carried out to determine the trends in iron deficiency. Of five studies that estimated serum transferrin receptor and/or serum ferritin levels, two studies were conducted in school settings, one in a garment factory, one in antenatal care centres in rural area, and one in a rural community setting (Table 13). [Iron deficiency was defined by serum ferritin (sFt) levels of <12 µg/L or <20 µg/L, and serum transferrin receptor (sTfR) levels of >8.5 mg/L or >5.0 mg/L.] Hyder *et al.* found an iron-deficiency prevalence of 42% (sFt <12 µg/L) among pregnant woman in rural areas (52). The prevalence of iron deficiency was 55% and 29% respectively for women who were anaemic (Hb <110 g/L) and who were non-anaemic. When sTfR was considered, 30% of the anaemic and 21% of the non-anaemic pregnant women had tissue iron deficiency with an overall prevalence of 25%. In one study, a prevalence of deficient iron store of 30% has been observed among school children aged 6-12 years (55). The high prevalence of deficient iron stores of 81% (sFt <12 µg/L) has been observed in anaemic (Hb 80-120 g/L) adolescents working in the garment factory (56). A recent study in a school setting has reported the prevalence of iron-deficiency status of 29.8% in 14-18 years age-group (57). A study conducted in a rural community found a prevalence of low iron status at 21.5% using a cut-off level for sFt <20 µg/L among school-age children of 6-15 years (50). However, when sTfR >5 mg/L was used for indicating tissue iron deficiency, the prevalence was 6.9%.

**Table 13:** Summary of prevalence data on iron deficiency in Bangladesh

Study period	Area	Setting	Age-group (years)	Sample size	Sampling design	Prevalence of iron deficiency (%)			Reference no.
						sFt (<12 μg/L)	sTfR (>8.5 mg/L)	sFt (<12 μg/L) and sTfR (>8.5 mg/L)	
1997	R	C-based ANC centre	14-44 (pregnant)	214	Cross-sectional	42	25	13	52
1998	R	S	6-12	164	Cross-sectional	30			55
2001	U	Garment factory	14-19 (anaemic, Hb: 80-120 g/L)	289	Randomized double-blind	81			56
2002	R	C	6-15	334	Cluster ran-domized	21.5*	6.9†		50
2003	R	S	14-18	178	Randomized double-blind	29.8			57

sFt <20 μg/L;

sTfR >5 mg/L; C=Community; ANC=Antenatal care; R=Rural; S=School; SFt=Serum ferritin; sTfR=Serum transferrin receptor; U=Urban

Based on the findings of all these studies, it can be concluded that, although iron deficiency is common in Bangladesh, it certainly does not totally explain the burden of anaemia. Further studies are needed to better understand the causes of anaemia. Iron fortification may help, but it will not solve the problem.

### Health consequences of anaemia

#### Child health outcomes

Hospital-based data principally from malaria-endemic regions have revealed that severe anaemia (Hb <50 g/L) in children is associated with an increased risk of death, but evidence for an increased mortality risk from moderate anaemia are inconclusive (59). Iron deficiency and anaemia are associated with poor cognition and motor development, and anaemic infants may continue to have poorer school achievement and behavioural problems in later childhood (60).

### Maternal health and pregnancy outcomes

There is evidence of a relationship between severe anaemia (Hb <47 g/L) and increased maternal mortality (61). However, the distinction between anaemia as a primary or a contributory factor in death is related to its acute or chronic pattern. Acute onset of severe anaemia (Hg <80 g/L) during pregnancy can lead to rapid cardiac decompensation and can be a primary cause of death relating to acute haemolysis due to an underlying disease, e.g. sickle-cell disease. Chronic anaemia, on the other hand, is considered to be a frequent contributory factor in death as a consequence of haemorrhage and infection. Iron-deficiency anaemia may contribute to increased morbidity and mortality by increasing maternal susceptibility to infection. However, evidence is scanty and inconsistent for the implication of moderate anaemia in excess maternal mortality or morbidity (61,62). There is some evidence of an association between maternal anaemia and low birthweight and preterm birth (63). However, Steer *et al.* reported a minimal association between low birthweight and anaemia with maternal haemoglobin values of 96-105 g/L (64). Other studies have reported other haemoglobin values as the minimal rate for low birthweight: 104-132 g/L in one study (65) and 105-125 g/L in Caucasian women in another study (66). A review of these studies pointed out that, during pregnancy, a haemoglobin value of <100 g/L is likely to reflect inadequate maternal nutritional status with respect to iron and other nutrients (63).

In a rural community in Bangladesh, an observational study has indicated a significant association of maternal haemoglobin levels of <80 g/L at first trimester and low birthweight (54). It may be predicted that 47.7% of pregnant women (Hg <80 g/L) in this study had inadequate nutritional status, including micronutrients, before pregnancy. Despite iron supplementation during pregnancy (73% good compliance), the prevalence of anaemia remained high. This study also suggested concomitant presence of other nutrient deficiencies, other than iron, and health risk factors.

### Haemoglobinopathies

Genetically-inherited disorders of haemoglobin or haemoglobinopathies may be causal factors for anaemia; however, their prevalence is among the least explored. Abnormal haemoglobin may be produced as a result of an alteration in the amino acid structure of the polypeptide chains of the globin fraction of haemoglobin, such as sickle-cell disease. In another form, the amino acid sequence is normal but the polypeptide chain production is impaired or absent for various reasons; these are thalassaemias. In Bangladesh, no screening programmes for thalassaemias are available; thus, there are no prevalence data regarding carrier status; however, a WHO report estimates that about 3% and 4% of populations are carriers of beta-thalassaemia and Hb-E-associated disease respectively in Bangladesh.

### Strategies to prevent anaemia

Three major interventions that may prevent anaemia include dietary diversification, food fortification, and supplementation. Dietary diversification involves promotion of a diet that contains a wider variety of naturally iron-containing foods with high bioavailability. Foods, such as meat, poultry, fish, and diary products, contain haem iron, which is more bioavailable than non-haem iron present in cereals, vegetables, and fruits. The principal diet of the people in poor countries consists mainly of cereals that are high in phytate, which is a known inhibitor of iron absorption. In many developing countries, the principal reason for iron-deficiency anaemia is poor dietary quality, and the intake of bioavailable iron is low. The diet of rural women of Bangladesh is principally based on cereal staples and has low iron and high phytate content (67). Efforts to improve dietary quality through home-gardening and animal husbandry will presumably increase the intake of many essential micronutrients; such programmes can also help combat poverty by generating income.

Fortification of a staple food item with iron is one of the effective strategies to prevent iron-deficiency anaemia in a population who regularly eat the staple food and, thus, increase their iron intake. Efficacy studies with iron salts (added to wheat flour) fortified with encapsulated ferrous sulphate and ferric pyrophosphate respectively demonstrated a reduction in the prevalence of iron-deficiency anaemia from 35% to 8% and 30% to 5% among Moroccan school children (68,69). Micronutrient ‘sprinkles’ is another way to provide home fortification of complimentary foods to reduce iron-deficiency anaemia in infants and young children (32). Fortification with iron is not universally successful, however, as demonstrated by a placebo-controlled efficacy trial conducted in a rural community by ICDDR, B in collaboration with MOST. A group of school-age children consumed wheat flour fortified with multi-micronutrients, including vitamin A and iron, with the primary objectives of improvement in iron status and haemoglobin and improvement in serum retinol levels. Although there was clear improvement in vitamin A status, there was no improvement in iron status or prevalence of anaemia (50).

The iron compound used in this ICDDR, B efficacy trial was hydrogen-reduced elementary iron. Being water insoluble and poorly soluble in diluted acid, the bioavailability of this form of iron is less than other forms, e.g. ferrous sulphate. A review of past studies has shown that the bioavailability of hydrogen-reduced iron was variable, 13-148% relative to ferrous sulphate; however, this may change depending on the particle size, or hypochlorhydria, or other causes of iron malabsorption (70).

However, a recent efficacy trial has reported that the RBV (relative bioavailability) of hydrogen-reduced iron is 49% in humans (71). It is conceivable that success of iron-fortification programmes depends largely on careful choice of the iron compound, dose, and other environmental factors. A recent WHO guideline recommended the following fortificants in order of preference: ferrous sulphate, ferrous fumarate, encapsulated ferrous sulphate, encapsulated ferrous fumarate, electrolytic iron (twice the level of ferrous sulphate), ferric pyrophosphate (twice the level of ferrous sulphate), and NaFeEDTA.

Iron-supplementation programmes aim to prevent anaemia or improve haemoglobin status in target population groups. The objective of such programmes in pregnant mothers is to improve anaemia status and its health consequences during pregnancy and the post-delivery period in mothers and their babies. Clinical trials have indicated the efficacy of iron supplementation in raising iron stores or haemoglobin levels (72). However, evidence is lacking that population-based iron-supplementation programmes have a notable impact on haemoglobin levels, iron status, or any other indices of maternal or perinatal health (73). The increased requirement of iron during pregnancy justifies supplementation for pregnant women where poor dietary intake cannot supply adequate iron during that period. However, in poor settings, the levels of micronutrients other than iron, including vitamin A, zinc, calcium, riboflavin, and vitamin B12, are also low in poor diets, and some of these micronutrients also contribute to anaemia. Thus, addressing only iron and/or folic acid may not be effective in correcting nutritional anaemia and may address only part of the problem concerning nutritional deficiencies. Therefore, where deficiencies of multiple micronutrients are common, a more appropriate formulation of multiple micronutrients may be considered (74). However, before such a programme is planned, aetiologies of anaemia, especially of nutritional origin in a particular area, need to be identified.

### Discussion

Anaemia is still a severe public-health problem in Bangladesh. Available data indicate that, over the last four decades, the situation has not improved. The cause of anaemia among young children is multi-factorial, including the low intake of bioavailable iron and high rates of infection. The intake of iron from complimentary foods is critical for the infant from six months as breastmilk alone cannot provide for the infant's increased need for iron for accelerated growth during that period. A WHO/UNICEF review of complimentary foods in developing countries concluded that requirements of iron might be difficult to meet from non-fortified complimentary foods, especially if animal products are not widely consumed. Home fortification of complimentary foods with sprinkles that contain iron plus other micronutrients could be an effective strategy to improve iron-deficiency anaemia in infants and young children. Community trials conducted in different countries, including Bangladesh, with sprinkles have demonstrated an impact in improving haemoglobin status in infants and young children.

Although iron deficiency is common, not all anaemias can be explained by iron deficiency alone. Iron-supplementation trials in controlled settings have shown to be efficacious in raising haemoglobin levels in pregnant women in Bangladesh and elsewhere. However, evidence is lacking that population-based iron-supplementation programmes have a notable impact on haemoglobin level and iron status or any other indices of maternal or perinatal health. In a rural community in Bangladesh, an observational study has indicated that, despite supplementation of iron during pregnancy to these women (73% good compliance), the prevalence of anaemia is still high. A significant association of maternal haemoglobin level of <80 g/L at first trimester and low birthweight was found in this study.

Fortification of a staple food item with iron could be an effective strategy to prevent iron-deficiency anaemia in this population who regularly eat the staple food and, thus, increase their intake of iron. Clearly, the choice of iron salt in the fortificant is critical when using this approach.

For anaemia, the lack of progress over 40 years makes one cautious about being optimistic for the success of the programme unless the aetiology of the problem is better understood. The perception about iron deficiency is common that iron fortification and iron supplements are a worthwhile recommendation wherever feasible. However, objective improvements may not be obvious. Thus, if anaemia is to be addressed as a serious issue, there needs to be much more basic research to understand the causes and physiology of anaemia in Bangladesh. Expecting iron supplements or fortificants to solve the problem is unlikely to be successful by itself.

Two evidence-based micronutrient interventions are now being proposed for widespread scaling up in Bangladesh: treatment of diarrhoea with zinc and the home-fortification of sprinkles micronutrient sachet. This will lead to questions from health planners and leading health professionals about their relative impact and the potential for programmatic interaction. Since these are leading nutritional programmes, these two interventions will need to be studied together and in combination.
